# Supplementary material for: Using smartphone-GPS data to quantify human activity in green spaces
Source: PLoS Comput Biol. 2022 Dec 15;18(12):e1010725. doi: 10.1371/journal.pcbi.1010725 (PMC9754188; doi:10.1371/journal.pcbi.1010725)
Supplement: S1 Appendix — (DOCX) [file pcbi.1010725.s001.docx]

**S1 Appendix – Excluding high activity areas**

The grid-cells provided by Mapbox span 100 x 100 meters and thus many on the perimeter of green spaces overlap with other features of land development. When that overlap includes areas of high activity, such as transportation routes in cities, using these grid-cells will inflate the activity values that are found within green spaces. One solution would be to only select grid-cells that occur entirely within the boundaries of a green space, but the irregular shape of these properties would result in a large exclusion of grid-cells. Moreover, the property boundaries of many greenspaces may be situated in a larger green belt or natural area meaning the area adjacent to a property is still representative of the area inside. A more systematic approach to exclude areas that are not representative of the green space is required. Selecting for overnight activity (12 – 6 am) is effective because many of these green spaces are closed overnight or have activity patterns that would fall below Mapbox threshold values (e.g., the parks are officially closed, there are no lights). Excluding grid-cells with high overnight activity removes almost half of the total number of grid-cells (Figure S1.1), although this includes more grid-cells than if selecting for only those that exist entirely within the green space boundary. The distribution of activity patterns between the excluded and included grid-cells are very similar, but the removed grid-cells had, on average, higher activity patterns (mean activity included = 0.034, excluded = 0.044). These excluded grid-cells were commonly along highways, major roads, or dense residental areas (for examples see Figure S1.2).


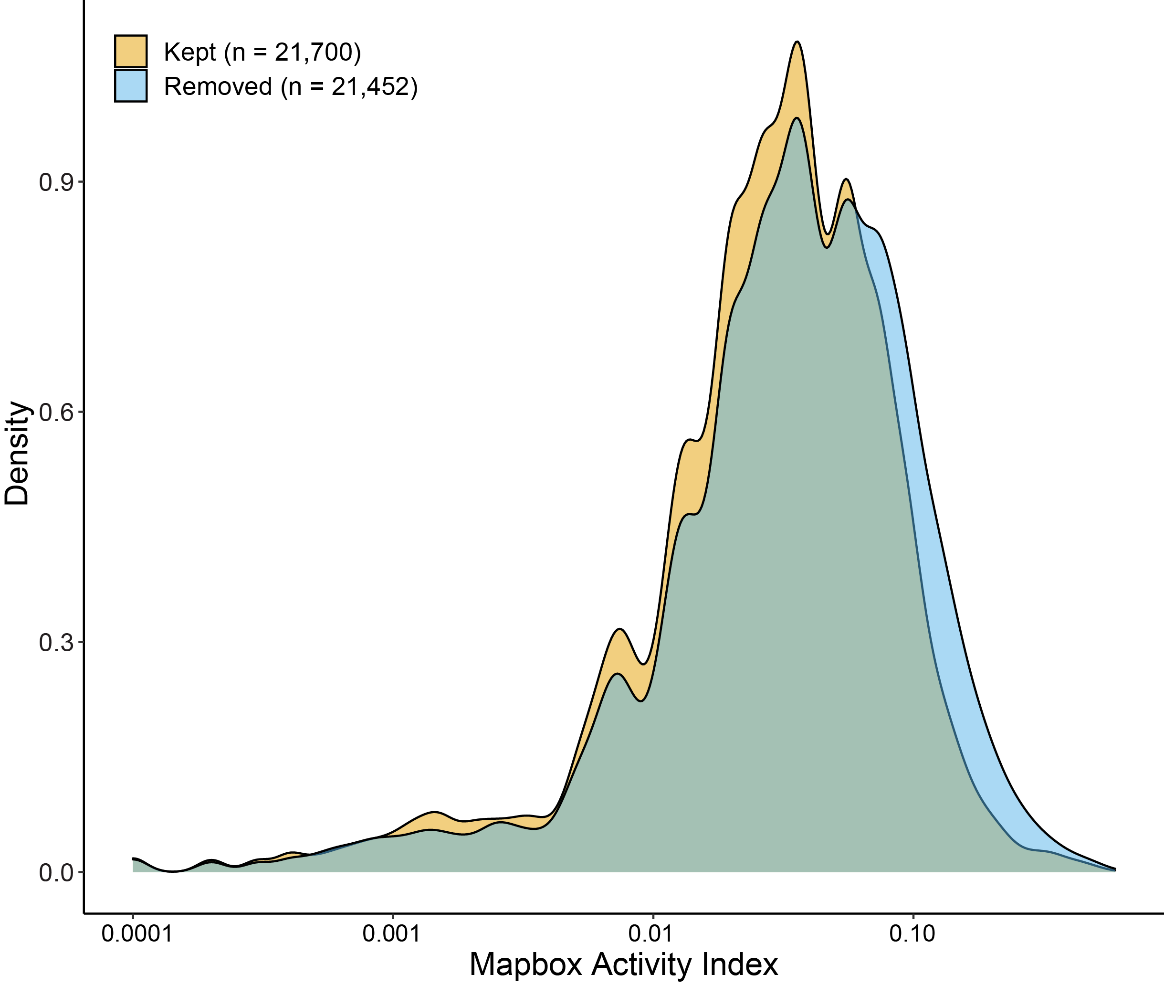


**Figure S1.1:** Distribution of Mapbox activity index values for the grid cells included (kept) in our analyses and those removed.

| **A** | **B** |
| --- | --- |
| **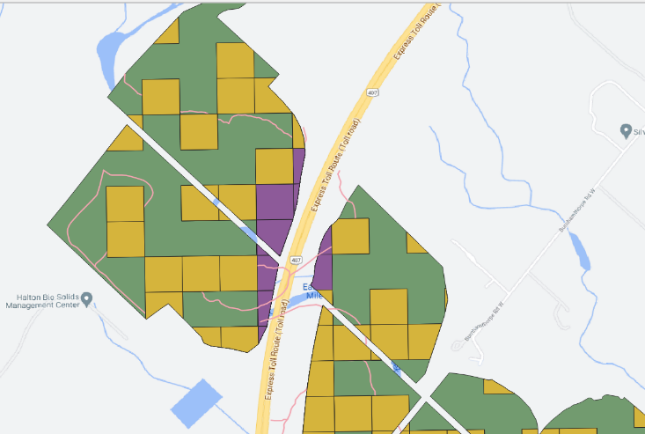** | **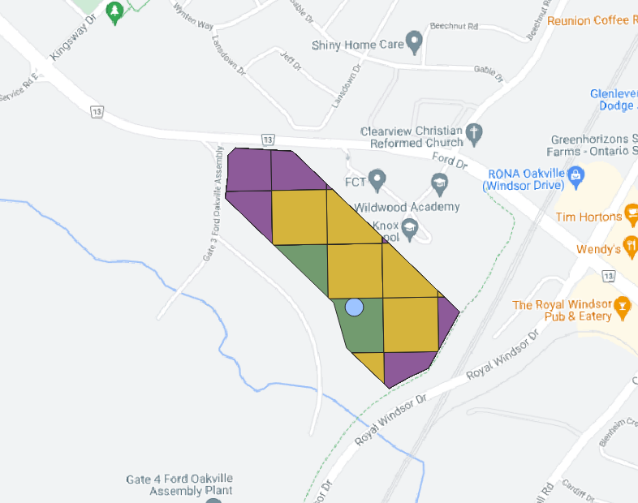** |
| **C** | |
| 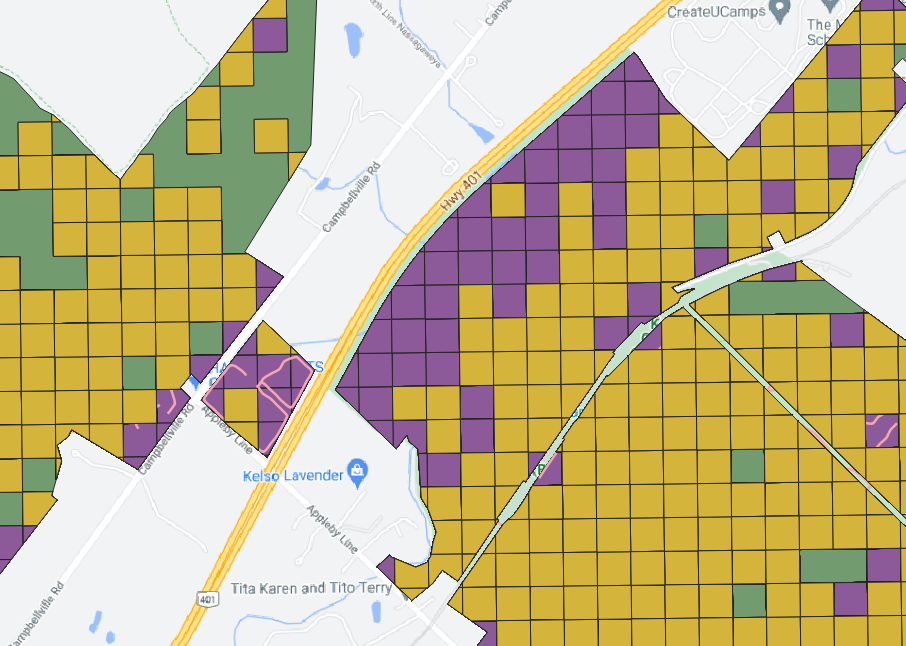 | |
| **Figure S1.2:** Each grid-cell represents 100 x 100 m of activity data. Purple grid cells are those identified to have high activity overnight. Yellow represents the Mapbox grid cells included in the estimation of human activity for green spaces. Green are areas within the green spaces where no activity data was recorded. Pink lines represent some of the trail network. Maps were created using Open Street Maps (https://www.openstreetmap.org/). | |
