# Supplementary material for: Using smartphone-GPS data to quantify human activity in green spaces
Source: PLoS Comput Biol. 2022 Dec 15;18(12):e1010725. doi: 10.1371/journal.pcbi.1010725 (PMC9754188; doi:10.1371/journal.pcbi.1010725)
Supplement: S1 Table — (DOCX) [file pcbi.1010725.s002.docx]

**Table S1:** A sample of the Mapbox movement data that includes columns for weekdays (agg_day_period), the 2-hour time window (agg_time_period), the month where the data has been summarized (month), a unique identifier for the respective grid cell, the minimum latitude and longitude of the grid cell (bounds), the centroid coordinates (xlat, xlon), and the proprietary measure of smart device activity (activity_index_total).

| **agg_day_period** | **agg_time_period** | **month** | **geography** | **bounds** | **xlat** | **xlon** | **activity_index_total** |
| --- | --- | --- | --- | --- | --- | --- | --- |
| 0 | 5 | 2020-06-01 | 30223132210132100 | -79.907684, 43.297198,  -79.906311, 43.298197 | 43.2977 | -79.907 | 0.372642 |
| 1 | 8 | 2020-06-01 | 30223132210132100 | -79.801940, 43.319183,  -79.800562, 43.320182 | 43.31968 | -79.8013 | 0.28166 |
| 0 | 6 | 2020-06-01 | 30223132210132100 | -79.800567, 43.308191,  -79.799194, 43.309191 | 43.30869 | -79.7999 | 0.311091 |
| 0 | 8 | 2020-06-01 | 30223132210132100 | -79.962615, 43.505732,  -79.961242, 43.506728 | 43.50623 | -79.9619 | 0.290336 |
| 1 | 7 | 2020-06-01 | 30223132210132100 | -79.801940, 43.319183,  -79.800567, 43.320182 | 43.31968 | -79.8013 | 0.247048 |
