# Supplementary material for: Using smartphone-GPS data to quantify human activity in green spaces
Source: PLoS Comput Biol. 2022 Dec 15;18(12):e1010725. doi: 10.1371/journal.pcbi.1010725 (PMC9754188; doi:10.1371/journal.pcbi.1010725)
Supplement: S2 Table — (DOCX) [file pcbi.1010725.s003.docx]

**Table S2:** The characteristics of all the green spaces within Conservation Halton that were explored during this study. Longitude and latitude correspond with the centroid of the green space boundaries. All properties requiring a reservation for entrance are classified as managed.

| **Name** | **Longitude** | **Latitude** | **Area Type** | **Managed** | **Perimeter (km)** | **Area (ha)** |
| --- | --- | --- | --- | --- | --- | --- |
| Administration | -79.9035 | 43.4229 | Other | Non-Managed Land | 1.101 | 3.1918 |
| Albert | -80.0825 | 43.3841 | Reserve Area | Non-Managed Land | 2.646 | 31.0222 |
| Andrusyshyn | -80.0806 | 43.43 | Reserve Area | Non-Managed Land | 1.221 | 7.5075 |
| Bronte Gorge | -79.9401 | 43.4075 | Natural Area | Non-Managed Land | 2.851 | 30.5541 |
| Burlington Beach | -79.8001 | 43.3107 | Conservation Area | Non-Managed Land | 5.889 | 8.8128 |
| Campbellville | -79.9827 | 43.4856 | Conservation Area | Non-Managed Land | 0.978 | 1.2917 |
| Carlisle | -79.975 | 43.396 | Conservation Area | Non-Managed Land | 7.975 | 27.7637 |
| Clappison Woods | -79.9002 | 43.3159 | Conservation Area | Non-Managed Land | 9.862 | 85.5145 |
| Cockshutt | -79.8315 | 43.3073 | Other | Non-Managed Land | 0.303 | 0.4281 |
| Crawford Lake | -79.9475 | 43.4657 | Conservation Area | Managed Land | 15.717 | 353.3575 |
| Croft | -80.0242 | 43.4123 | Reserve Area | Non-Managed Land | 2.378 | 34.3269 |
| Escarpment | -79.9561 | 43.5344 | Natural Area | Non-Managed Land | 6.623 | 30.5494 |
| Esquesing | -79.948 | 43.5398 | Natural Area | Non-Managed Land | 1.622 | 15.2311 |
| Forest Creek Estates | -79.9053 | 43.4279 | Natural Area | Non-Managed Land | 3.882 | 15.396 |
| Forster & Burt | -79.6721 | 43.4477 | Other | Non-Managed Land | 1.964 | 5.3375 |
| Fuciarelli | -79.9981 | 43.3388 | Reserve Area | Non-Managed Land | 4.867 | 60.2088 |
| Glenorchy | -79.7777 | 43.4623 | Conservation Area | Non-Managed Land | 34.661 | 404.4995 |
| Grindstone Creek | -79.8853 | 43.3169 | Natural Area | Non-Managed Land | 6.179 | 63.7958 |
| Guelph Junction | -80.0031 | 43.4689 | Reserve Area | Non-Managed Land | 2.882 | 28.2019 |
| Hager Rambo | -79.8173 | 43.3309 | Other | Non-Managed Land | 9.239 | 10.768 |
| Hilton Falls | -79.9755 | 43.5147 | Conservation Area | Managed Land | 21.468 | 655.4967 |
| Kassam | -80.0585 | 43.5089 | Reserve Area | Non-Managed Land | 1.934 | 16.6238 |
| Kelso | -79.9366 | 43.5037 | Conservation Area | Managed Land | 19.903 | 459.5864 |
| Kerncliff | -79.8569 | 43.3497 | Conservation Area | Non-Managed Land | 5.574 | 37.3252 |
| Kilbride | -79.9579 | 43.442 | Natural Area | Non-Managed Land | 10.405 | 126.2242 |
| Kiwanis | -79.9991 | 43.5389 | Natural Area | Non-Managed Land | 2.596 | 41.2202 |
| Knight | -80.0705 | 43.4048 | Reserve Area | Non-Managed Land | 2.681 | 18.5708 |
| Lake Medad | -79.8913 | 43.3665 | Reserve Area | Non-Managed Land | 2.094 | 26.9479 |
| Medad Valley | -79.9042 | 43.3926 | Reserve Area | Non-Managed Land | 1.831 | 9.6038 |
| Middletown | -80.062 | 43.3689 | Reserve Area | Non-Managed Land | 0.602 | 2.124 |
| Milton | -79.877 | 43.5136 | Other | Non-Managed Land | 6.514 | 4.8001 |
| Moffat-Badenoch Swamp | -80.0735 | 43.4804 | Natural Area | Non-Managed Land | 10.018 | 160.5223 |
| Morrison-Wedgewood | -79.6881 | 43.466 | Other | Non-Managed Land | 9.607 | 22.1411 |
| Morriston | -80.12 | 43.4466 | Other | Non-Managed Land | 0.404 | 0.7638 |
| Mount Nemo | -79.8772 | 43.4193 | Conservation Area | Managed Land | 17.672 | 204.1112 |
| Mountsberg | -80.0389 | 43.4634 | Conservation Area | Managed Land | 26.114 | 556.7478 |
| Nelson Escarpment Woods | -79.8603 | 43.3636 | Natural Area | Non-Managed Land | 1.939 | 5.3288 |
| Ontario Heritage Trust | -79.9591 | 43.4451 | Natural Area | Non-Managed Land | 10.205 | 87.5001 |
| Paiement | -80.0473 | 43.4284 | Reserve Area | Non-Managed Land | 1.589 | 11.5533 |
| Plaikner | -79.939 | 43.601 | Reserve Area | Non-Managed Land | 2.114 | 22.3643 |
| Pleasantview | -79.9122 | 43.2961 | Natural Area | Non-Managed Land | 13.154 | 57.1412 |
| Queen Elizabeth | -79.6927 | 43.4511 | Other | Non-Managed Land | 1.159 | 4.093 |
| Rattlesnake Point | -79.9289 | 43.4761 | Conservation Area | Managed Land | 17.344 | 313.4842 |
| Robert Edmondson | -80.0315 | 43.4898 | Conservation Area | Managed Land | 2.556 | 30.9579 |
| Roberts Reed | -80.035 | 43.5478 | Reserve Area | Non-Managed Land | 2.641 | 33.4411 |
| Scotch Block | -79.9478 | 43.5726 | Other | Non-Managed Land | 4.925 | 33.23 |
| Shanahan | -80.0435 | 43.4875 | Natural Area | Non-Managed Land | 3.959 | 68.514 |
| Sixteen Valley | -79.7759 | 43.5016 | Natural Area | Non-Managed Land | 2.3 | 30.6336 |
| Speyside | -79.9746 | 43.575 | Natural Area | Non-Managed Land | 2.676 | 26.1658 |
| Stewart | -79.7463 | 43.4577 | Conservation Area | Non-Managed Land | 2.31 | 20.8627 |
| Tirion | -79.9627 | 43.5575 | Natural Area | Non-Managed Land | 1.964 | 20.1398 |
| Waterdown Woods | -79.8664 | 43.338 | Conservation Area | Non-Managed Land | 13.1 | 120.1421 |
| Wildflower Woods | -79.6587 | 43.4894 | Natural Area | Non-Managed Land | 1.434 | 9.7133 |
