# Supplementary material for: Using smartphone-GPS data to quantify human activity in green spaces
Source: PLoS Comput Biol. 2022 Dec 15;18(12):e1010725. doi: 10.1371/journal.pcbi.1010725 (PMC9754188; doi:10.1371/journal.pcbi.1010725)
Supplement: Attachment — Submitted filename: Response letter to reviewers 2022_Oct 7.docx [file pcbi.1010725.s004.docx]

October 7, 2022

Dear Editorial Board of PLoS Computational Biology

Thank you for the opportunity to resubmit our manuscript: **Using smartphone-GPS data to quantify human activity in green spaces.** We have incorporated the comments from the three reviewers. Each reviewer positively reviewed the paper, describing it as an early study using anonymized GPS data from smartphones to better understand human-nature interactions. The reviewers were thorough in their comments raising concerns that the methodology was vague, the framing around novelty was overstated, and that more consistent terminology could be used. These were useful suggestions and we have carefully revised our manuscript to address all the comments. Specifically, we significantly expanded our methods, provided additional results, and revised terminology throughout to be more consistent. We believe these revisions substantially improved our manuscript for future readers of PLoS Computational Biology and thus were happy to incorporate them.

All coauthors fully participated in and accept responsibility for the manuscript. The manuscript has not yet been published and is not being considered elsewhere for publication. We hope that you enjoy the revised manuscript and look forward to hearing back from you.

Sincerely,

Alessandro Filazzola on behalf of all co-authors

Reviewer's Responses to Questions

**Comments to the Authors:
Please note here if the review is uploaded as an attachment.**

Reviewer #1: The authors provide an interesting analysis of human activity in green area, a subject that is of major importance in the perspective of designing cities that allow for open air activity, especially in pandemic times.
In general the text seems well structured, but it is hard to grasp the meaning of the work carried out by the authors by reading the results and discussion sections.
The interpretation of the results is placed outside of the discussion section, which makes it look like something less important, while actually it is the main key to understand all the results.
The description of the methods is too vague and general, it is never clear if the metrics are measured using a specific month or a temporal average and if seasonality is taken into account.
In the text the authors often refer to their mobility metrics as "mobility data", which is misleading and hinders any understanding of the quantities at play.
Most of the figures only show the results for a sub-set of the total number of green areas present in the dataset (53), but the authors never justify this choice.
I think the authors need to put more effort in the description and justification of the methodology and results in the text, correcting the lexicon and making clear what are the magnitudes measured from the mobility data. For this reason I suggest major revision.

Thank you for the positive feedback on our manuscript. We agree that some of our methods or descriptions of analyses were vague. We have since revised our manuscript to be more specific, including using a more intuitive metric of Mapbox activity data, expanding our methods to better document our actions, and provide more details in our results. Specifically, we made clear the time frames that we used, explained why subset of green spaces are used for each analysis, and address all of your comments provided below. Thank you for your suggestions in improving out manuscript.

Here a list of major concerns:

- repetition in line 63 wrt 61

Revised

- line 115: “By contrast, biodiversity data are rarely collected hourly” it is not clear what type of biodiversity data do the authors expect to collect at a hourly time scale.

Revised on lines 206-207.

- Fig2: not clear what is the temporal scale used in the figure, e.g. a day, one week, one month, an average of months?

Revised, see figure 2.

- line 197: typo “gereen”

Corrected

- Fig3: plots need correlation scores and pvalues to be shown in the figure

Revised. We have added co-efficient values and pvalues to the figure caption for this figure and Figure 1.

- line 203: “Human activity varied considerably by ELC-CC. Forest and cultural land classes were
204 associated with the most mobility data, followed by talus and cliff” what is the meaning of being associated with the most mobility data? what metric of mobility are you considering here?

Revised on lines 201-206.

- line 207: “By contrast, forests and cultural were used relatively infrequently in proportion to their
abundance among green spaces” is this showed in Fig4B? it was not clear by looking at the picture nor by reading the caption that frequency of visiting was measured by this metric. Indeed I failed to grasp the real difference between Fig.4A and B. Also, again it is not clear what temporal scale are the authors using to measure mobility here.

We apologize for being unclear and not more explicitly describing the differences between the two figures. We have revised the text and expanded the figure caption to be clearer. See figure caption for Figure 44 and lines 201-206.

- Fig5: it is not clear what are the causal hypotheses that are assumed in this analysis. It is interesting to check correlations between different metrics of human activity and trees diversity, however the authors do not explain what are the known hypotheses regarding the influence of human activity on trees diversity in green areas.

We have expanded in the introduction the different hypotheses in which human activity and biodiversity may correlate. See lines 84-92.

- i do not understand why the interpretation of results composes a different section from the discussion. itwas hard to read and grasp the meaning of all analyses and results until a paragraph after discussion finally explained the reasoning behind the hypotheses tested in the results section.

It is typical for manuscripts in PLoS Computational Biology to have separate Results and Discussion sections. While these two sections may be combined, we believe the separate sections help to separate pattern description from pattern inference.

- what is the spatial resolution of biodiversity data? I assume they are aggregated at level of green spaces

Thank you for the suggestion, we have added it now. See lines 507-508.

- line 421: there are 53 green spaces but only 5 are shown in Fig1 and only 11 in Fig.3, how come the authors discarded the rest of green areas from the analyses?

Not all had data that was available for each of the analyses. We have added text to make this limitation more explicit throughout the MS and also listed on lines 480-482.

- the authors often refer to the two mobility metrics showed in equation 1 and 2 as “mobility data”. it really hinders the understanding of the analyses and makes the reasoning of the work much more obscure than it could be. The authors need to rephrase most of the sentenced including “mobility data” to correctly point at the metrics they are referring to when describing the results. Mobility data per se does not correlate, it is the metrics that we extract from them to be quantitatively comparable and measurable. For instance “For every green space, we calculated the total adjusted mobility data” this sentence has no meaning, one thing is to say that mobility data is very informative on human activity, the other is to proper measure some quantities from it. Please rephrase every part in which you refer to mobility metrics, properly using the terms associated with the two metrics that you compute in the Methods section.

Great suggestion thank you! We have now standardized our language when referring to our calculating metrics as “activity density” and “activity coverage” to represent the two metrics calculated in equations 1 and 2. We believe this will better differentiate our patterns and assist readers in understanding our results.

- the activity density metric is calculated using the activity provided by Mapbox, this dataset provides the normalized activity in each grid cell of the given green area. Is the activity normalized on the maximum or is it normalized to have the sum of all cells activities = 1? In case it is the second one, this activity in a park with a lot of activity in all the grid cells would provide a very low score.

We apologize for being unclear. Mapbox does have a systematic workflow for normalizing the data across the country and scaled to a baseline value. We have expanded our methods to more explicitly detail the workflow Mapbox takes to process the data. See lines 411-443.

- the activity density metric has the green area surface at the denominator, does it include all the green area surface or only those cells with a minimum activity required to appear in the dataset?

The denominator represents all the green space area, not just those that appeared in the dataset. We expanded on these details on lines 515-527.

- In general the authors need to put more effort in the description of the mobility metrics that they design, in order to correctly address the quantitites at play and the temporal scales. For example, the activity metric is the sum of all cells activities divided by the green area total surface, is this sum running over grid cells only or also on time?

We apologize for not being clearer in our description of the temporal scales. We have revised throughout the manuscript and in the figure captions to emphasize that the values we are reporting are summarized across the three months. We have also revised our calculation of the mapbox metric data into one that is more clearly understandable.

Reviewer #2: This manuscript reports the integration of different analytical methods to connect the visitation patterns deduced from mobile phone data to the pathways, land use and species in urban green areas. Although the article is interesting and well-written, I feel that the approach is not too innovative (see for example Le Normand et al, PLOS ONE, 2014).

Thank you for the positive feedback! Throughout our manuscript we have revised the text to refrain from mentioning this as the first study of its kind and tempered language of novelty. Instead we have rephrased this as an early study testing a novel dataset (i.e., Mapbox activity data) and relating it to green space features.

Additionally, the data is not properly described, which difficults the understanding of the quantitative results shown in this manuscript, with only a partial description included after the results. In particular, I suggest the authors to strengthen the following points:

We apologize for not having clearer description of the methods. We have expanded the methodology to include more details about how the activity index is calculated, the temporal resolution that is involved, specificity around the metrics being used, and some other specific suggestions provided by yourself and the other reviewers.

-The mobility is quantitatively introduced in the manuscript, but the used variable is not explained (for example, what is the y-axis in Fig. 1? After log-transforming, I find remarkable that any variable has the value 140). Authors should then explain accurately first what the mobile cell data is, whether call detailed records, high-resolution trajectories..., and secondly this mobility is measured as number of unique users, number of calls, number of points falling on a grid cell, cumulative time spent on a grid cell... There are many options, so I strongly recommend to specify what is measured.

Good point. We agree our original metric of activity was slightly convoluted and have instead opted for a simpler version that users the sum activity index provided by Mapbox (a proportion between 0-1) multiplied by the adjusted area of the grid-cell (based on overlap with greenspace) and then summed across the entire green space polygon and divided by the area of the polygon. The activity density metric thus more closely resembles a proportion of human activity in the green space. We have revised our figures, results, and methods to use this more simplified measure that is not log-transformed.

-Fig 1: I recommend using data points, apart from the letters, to know specifically where the point is with respect to the fit. What is the formula of these fits and their results? I find interesting that the slopes are different for weekdays and weekend, and think that some comments on this should be included in the main text.

We have added the statistics around the slopes and have removed the labels identifying each of the sites, replacing them with points. We have added lines joining the same sites that have weekend and weekday values. See revised figure 1.

-L162: this 9.2% higher should be commented. I guess that this is the total mobility after aggregating all the parks. However, this number does not make too much sense for me, because the curves on Fig. 1 are not parallel in a semilog scale. First, I would be happy to see if they are in the log-log scale, and then mentioning a % excess would make sense. Otherwise, it does not make sense, because including parks with higher or lower mobilities would impact this number.
We have removed the log-transformation throughout making this comparison more appropriate.

-L177: Fig. 1 is referenced here, but I think this reference should point to Fig. 2.

That is correct, thank you for identifying it!

-Fig. 2: it would be nice to see some text specifying which is Hilton Falls and which is Kelso Conservation Area.

Great suggestion. We have added the text identifying each property in the figure.

-L203: use of an acronym not previously introduced, ELC-CC.

We have defined the acronym here.

-Fig 3: need to specify the fitted formulas and the results.

We have added the fitted formulas to the figure caption.

Reviewer #3: Summary of the research and overall impression


This paper investigates associations between human occupancy and ecological characteristics of parks in the Greater Toronto Area using a new data resource: aggregated grid-cell mobility data. This case study is an example of one way these data can be applied to understand relationships between park features and patterns in human use. The paper has two areas of contribution: the first is a methodological example for using human mobility data in park management and human-nature research. It also provides a series of findings describing associations between human occupancy and park features, from a sample of 53 parks. This is a novel and compelling contribution to the literature as it is a very early example for relating mobility data to park and ecological features, and probably the first to do so using specifically mapbox data, and other ecological data such as park trails or tree diversity. This paper provides an example for park managers and municipal organisations looking for ways to apply mobility data in management of green amenities. The strength of the paper is in its clarity and systematic study of park usage, and its description of the approach taken for using and preparing the raw mobility data for use. The paper can use some refinement in how it presents related literature, and more clarity in how it structures and portrays the overall contribution.

Thank you very much for the positive feedback on our manuscript and for the helpful comments provided below

Evidence and examples

My first and main comments are focused on the Abstract but could relate to the introduction and discussion. These have to do with clarifying the purpose of the paper. It is unclear from the outset if the purpose is to provide methods or results or your models and data. Of course, including both is fine, but the methods-based contribution does not align with your research questions, and does not receive a strong literature component in the discussion or the introduction. I don't see any review of other studies that use mobility data in recent literature. In the abstract you state this is the first time mobility data has been used to quantify human activity in green spaces. This is not the case. Although, it is definitely an early study with important and novel contributions. In addition, you state you provide a framework for mobility data usage. I would soften this term as the term “framework” suggests something more formal than an outline of your own process. Ideally, your approach and recommendations could be highlighted with a summary, table or figure in the discussion as I expect these will be interesting and useful to readers.

In line with my above comments, I agree that your methods will likely be very useful for planners, or park managers, although there was no study conducted with end users of this method nor an analysis of the process compared with other methods. Therefore, one option could be to present the methods as a reflection of your own personal experiences doing the analysis for this study, with some helpful tools (such as R script) and the findings of the study as the core contribution. But the opposite could be the case, where a method is presented (but would include a more systematic methods comparison).

These are great suggestions, thank you. We agree that the manuscript could be more explicit in its intentions and overall objectives. We have added an specific purpose into the abstract and throughout the manuscript detailing how our paper is intended to a) describe how GPS data from smart devices can be used in human-nature research and b) define associations between park features and human activity. Additionally, we have softened terminology such as framework and revise the manuscript to be more from our perspective in doing these analyses rather than a heuristic to adopt. These changes have been made throughout the manuscript, but notable we have provided more explicit intensions on these lines 24-26 and 124-127.

This issue also relates to Table 1, which I believe to be a useful representation of the challenges related to using mobility data and a helpful tool for readers. Although it is not placed within the context of other literature that review these same topics or apply similar data. If the table is in the introduction it should have citations. If you choose to relocate it in the discussion you could frame it specifically around your study and specific data type.

Thank you for this suggestion. We have moved table 1 into our discussion and rephrased the text of the challenges from our perspective and in the content of green spaces. Using many of the citations you have provided below, we have better framed this issues with the current literature. See limitations sections on lines 286-337.

To help here are a few papers that have covered similar topics and could help contextualise your work (as well as observations made in Table 1).
These are great citations thank you! We have incorporated many of them into our writing.

Moro, E., Calacci, D., Dong, X. et al. Mobility patterns are associated with experienced income segregation in large US cities. Nat Commun 12, 4633 (2021). [https://doi.org/10.1038/s41467-021-24899-8](https://can01.safelinks.protection.outlook.com/?url=https%3A%2F%2Fdoi.org%2F10.1038%2Fs41467-021-24899-8&data=05%7C01%7Ckbarrett%40hrca.on.ca%7Cea789165a5af4fe7f42808da705271bb%7Cf54623c78bce4eaf9bc3d671d530b7fe%7C0%7C0%7C637945796685813347%7CUnknown%7CTWFpbGZsb3d8eyJWIjoiMC4wLjAwMDAiLCJQIjoiV2luMzIiLCJBTiI6Ik1haWwiLCJXVCI6Mn0%3D%7C3000%7C%7C%7C&sdata=8bkJUNNfEItShOq6tCTMzkmTgx7YroNxrVOTiaiszBs%3D&reserved=0)

Saxon, J. (2021). Empirical measures of park use in american cities, and the demographic biases of spatial models. Geographical Analysis, 53(4), 665-685.

Song, Y., Newman, G., Huang, X., & Ye, X. (2022). Factors influencing long-term city park visitations for mid-sized US cities: A big data study using smartphone user mobility. Sustainable Cities and Society, 80, 103815.

Song, Y., Huang, B., Cai, J., & Chen, B. (2018). Dynamic assessments of population exposure to urban greenspace using multi-source big data. Science of the Total Environment, 634, 1315-1325.

Venter, Z. S., Barton, D. N., Gundersen, V., Figari, H., & Nowell, M. (2020). Urban nature in a time of crisis: Recreational use of green space increases during the COVID-19 outbreak in Oslo, Norway. Environmental research letters, 15(10), 104075.

Yoo, E. H., & Roberts, J. E. (2022). Static home-based versus dynamic mobility-based assessments of exposure to urban green space. Urban Forestry & Urban Greening, 70, 127528.

Korpilo, S., Virtanen, T., & Lehvävirta, S. (2017). Smartphone GPS tracking—Inexpensive and efficient data collection on recreational movement. Landscape and Urban Planning, 157, 608-617.

Kim, J., Thapa, B., Jang, S., & Yang, E. (2018). Seasonal spatial activity patterns of visitors with a mobile exercise application at Seoraksan National Park, South Korea. Sustainability, 10(7), 2263.

Ladle, A., Galpern, P., & Doyle-Baker, P. (2018). Measuring the use of green space with urban resource selection functions: An application using smartphone GPS locations. Landscape and Urban Planning, 179, 107-115.
Vanky, A. P., Verma, S. K., Courtney, T. K., Santi, P., & Ratti, C. (2017). Effect of weather on pedestrian trip count and duration: City-scale evaluations using mobile phone application data. Preventive medicine reports, 8, 30-37.

Rout, A., Nitoslawski, S., Ladle, A., & Galpern, P. (2021). Using smartphone-GPS data to understand pedestrian-scale behavior in urban settings: A review of themes and approaches. Computers, Environment and Urban Systems, 90, 101705.


Related to the above issues, I would also edit your research questions #2. (line 141). I do not believe you cover the question as written. From my readings the question would be better if it related to determining how the anonymized mobility data intersected with the ecological land classification Community Class.

We have revised question #2 to speak directly to our experience working with anonymized location data. This change aligns with our expanded discussion on the challenges associated with using location data in green spaces and the moving of table 1 to the discussion.

Lines 21-22 Consider the terminology you are using in the title (and possibly) throughout. You start with the term: “mobile cellular data”. I realise this term is appropriate because data is provided as a cell grid - but it could be misinterpreted as data that is aggregated at the cell tower level. Cell tower data has been available for decades and the term may not represent the novel nature of your work. Mapbox provides data that is structured differently than other data providers: where data is aggregated by polygon or point of interest. It is also unique in that it provides a relative rating for visitation rather than raw numbers. I don't know if there is a specific term for this kind of data but I believe the literature should begin to differentiate between mobility data with clear language as it can become confusing, especially for those aiming to compare options. To cover data from mobile phones that infers human travel information the term “mobility data” is often used, although it may not apply to your work as the data does not include movement or travel information, but rather occupation counts for a cell grid. I recommend you review some of this literature and describe your terms and their definitions in the introduction.

This is an interesting point. We have reviewed the literature and “mobility data” is certainly one of the more commonly used terminologies. However, we agree that mobility data may be confused with mobile (as in movement data) or mobile as in mobile phone. Another common term is “smartphone GPS locations” that is more accurate but excludes other devices with location services. Thus, we have revised our definition to be “anonymized GPS data from smart devices”. Throughout our manuscript we have revised mention of mobility data to either refer to the specific metric we calculated in our results (e.g., adjusted activity density), “human activity data” more generally, or when specifically describing our approach “GPS data from smart devices”.

It would be helpful to include a description of the landscapes that the study park sample is located in, and citations relating to challenges with management of the parks with these specific ecological or social features in the introduction. Please also indicate earlier in the paper that your sample includes 53 parks, (or 7 for part of the study).

Thank you for the thoughtful comment. We have expanded a paragraph in the introduction to include a description of the landscapes regarding both the ecological and social features that the study park sample is located in and have included the citations. Revised on lines lines 106-122.

Line 155, please refer here to where in your own document (or elsewhere) that threshold levels are defined by mapbox?

We have expanded significantly on the methodology used by Mapbox to calculate their activity index. See lines 410-442.

I find FIgure 5 almost impossible to read due to overprinting of text. Is there another visual type that would make these findings more readable (this also applies to figure 3, although less of an issue)

We have revised figures 1 and 3 to no longer have green space labels to improve legibility. We have also revised figure 5 to be more readable by increasing the space between labels.

Line 324-325 I think the causation or mechanistic relationship issue would be less of a risk to readers if the sample was more clearly explained earlier - particularly in reference to other parks or landscape types. For example - rock formations are not available in many other areas, and thus, these findings would not be transferable. Although I think a sample of 53 is large enough to provide some generalizability across similar landscapes. Just how general your findings can be would be useful to include here. That said, I think your discussion of the reason for correlations is helpful and clear.

This is a good suggestion and one also suggested by reviewer #1. We have moved into the introduction an explanation of the mechanisms that we expect human activity and the environment to correlate. We believe this better sets up our third research question and our interpretation in the discussion.

Line 411 One clear advantage of grid-cell mobile data vs data cropped by a polygon boundary is the ability to isolate specific locations near other confounding features. I think the method you use to filter out noise from highways and other non-park uses, is very helpful. The graphic you provide in supplemental materials showing highway locations and nearby cells is useful but I think you should also include more information in the main paper, particularly how much data was removed, the distribution of raw data vs the filtered data and number of removed cells or rows of data due to these changes. This is particularly important if you aim to position your paper as a methods-focused contribution.

We agree this is a powerful component of the grid-cell activity index. We have revised the focus of our manuscript away from a methods-focused contribute in response to your above comment. However, we agree providing some additional details about the filtering of grid-cells within green spaces would be useful for future readers. We have expanded on the supplemental section providing examples, detailing how much data was removed, displaying a figure of the distributions between the data, and a brief discussion on these patterns. See Supplemental S1.

Line 481-483 Can you clarify the verbiage here? As a reader I am getting confused about which term refers to the traditional visitation estimates vs visitor counts from the mobility data.

Revised on lines 381-382.

Line 481 - 483, Can you please include models for more advanced linear models, i.e. those that include interactions or multiple variables?

We have added more details throughout our manuscript about the effect sizes, error, degrees freedom, F-statistics, and p-values for all of our linear models.

Line 509 - your Github link does not currently open

The Github link will be made publicly available on manuscript acceptance

Please provide summary statistics for raw and cleaned data. Particularly for Mobility data which I believe is not being shared.

We provide the summary data in an appendix file with this revision submission and will make the Github repo code all publicly available upon publication.
