# Supplementary material for: Using smartphone-GPS data to quantify human activity in green spaces
Source: PLoS Comput Biol. 2022 Dec 15;18(12):e1010725. doi: 10.1371/journal.pcbi.1010725 (PMC9754188; doi:10.1371/journal.pcbi.1010725)
Supplement: Attachment — Submitted filename: Response letter to reviewers 2022.docx [file pcbi.1010725.s005.docx]

November 7, 2022

Dear Editorial Board of PLoS Computational Biology

Thank you for the opportunity to resubmit our manuscript: **Using smartphone-GPS data to quantify human activity in green spaces.** The reviewers appeared satisfied with our extensive review of the manuscript on previous submission. Reviewer 1 had two outstanding comments with respect to clarifying the number of devices associated with the human activity index and the percent area of human activity. We addressed both comments on lines 526-534. Thank you very much to the editorial team of PLoS Computational Biology and each of the reviewers for their invaluable feedback that improved our manuscript. We look forward to seeing this paper in publication.

All coauthors fully participated in and accept responsibility for the manuscript. The manuscript has not yet been published and is not being considered elsewhere for publication.

Sincerely,

Alessandro Filazzola on behalf of all co-authors
